# Reduced Claudin-12 Expression Predicts Poor Prognosis in Cervical Cancer

**DOI:** 10.3390/ijms22073774

**Published:** 2021-04-06

**Authors:** Abidur Rahman, Makoto Kobayashi, Kotaro Sugimoto, Yuta Endo, Manabu Kojima, Shigenori Furukawa, Takafumi Watanabe, Shu Soeda, Yuko Hashimoto, Keiya Fujimori, Hideki Chiba

**Affiliations:** 1 Department of Basic Pathology, Fukushima Medical University School of Medicine, Fukushima 960-1295, Japan; abidavid@yahoo.com (A.R.); makokoba@fmu.ac.jp (M.K.); yenyen@fmu.ac.jp (Y.E.); 2 Department of Obstetrics and Gynecology, Fukushima Medical University School of Medicine, Fukushima 960-1295, Japan; m2149@fmu.ac.jp (M.K.); s-furu@fmu.ac.jp (S.F.); t-wata@fmu.ac.jp (T.W.); s-soeda@fmu.ac.jp (S.S.); fujimori@fmu.ac.jp (K.F.); 3 Department of Diagnostic Pathology, Fukushima Medical University School of Medicine, Fukushima 960-1295, Japan; ykhykh@fmu.ac.jp

**Keywords:** claudin, CLDN12, gynecological cancer, tight junction, biomarker, squamous cell carcinoma, adenocarcinoma

## Abstract

Background: Within the claudin (CLDN) family, *CLDN12* mRNA expression is altered in various types of cancer, but its clinicopathological relevance has yet to be established due to the absence of specific antibodies (Abs) with broad applications. Methods: We generated a monoclonal Ab (mAb) against human/mouse CLDN12 and verified its specificity. By performing immunohistochemical staining and semiquantification, we evaluated the relationship between CLDN12 expression and clinicopathological parameters in tissues from 138 cases of cervical cancer. Results: Western blot and immunohistochemical analyses revealed that the established mAb selectively recognized the CLDN12 protein. Twenty six of the 138 cases (18.8%) showed low CLDN12 expression, and the disease-specific survival (DSS) and recurrence-free survival rates were significantly decreased compared with those in the high CLDN12 expression group. We also demonstrated, via univariable and multivariable analyses, that the low CLDN12 expression represents a significant prognostic factor for the DSS of cervical cancer patients (HR 3.412, *p* = 0.002 and HR 2.615, *p* = 0.029, respectively). Conclusions: It can be concluded that a reduced CLDN12 expression predicts a poor outcome for cervical cancer. The novel anti-CLDN12 mAb could be a valuable tool to evaluate the biological relevance of the CLDN12 expression in diverse cancer types and other diseases.

## 1. Introduction

Cervical cancer is the fourth leading cause of cancer-related deaths in women [1,2]. In 2018, it was estimated that more than half a million patients were diagnosed with cervical cancer, and that over 300,000 deaths occurred worldwide [3]. The most and second-most histological types of cervical cancers are squamous cell carcinoma (SCC) and adenocarcinoma (ADCA), which account for 70–80% and 15–25% of all cases, respectively [4,5]. In addition, the major risk factor for cervical intraepithelial neoplasia (CIN) and cervical cancer is persistent infection with high-risk human papillomavirus (HPV) strains, largely HPV16 and HPV18 [6]. The incidence and mortality of cervical cancer have been reduced in developed countries, due to organized screening programs [7]. Nevertheless, the 5-year overall survival (OS) for women with locally advanced cervical cancer is about 70% following chemoradiotherapy, and the prognosis for patients with recurrent or metastatic disease remains poor [2]. Along these lines, biomarkers that can distinguish cervical cancer cases with good and poor outcomes are required for patients to avoid both overtreatment and undertreatment, as well as to improve the quality of care.

The claudin (CLDN) family of tight-junction proteins functions not only as paracellular barriers or pores for selective ions and solutes, but also as signaling platforms to coordinate diverse cellular behaviors [8,9,10,11,12,13]. Concerning the signaling properties of CLDNs, we recently demonstrated that the CLDN6-adhesion signal regulates transcription factor activity [14]. In summary, we identified that CLDN6 couples with Src-family kinase (SFKs) in its extracellular loop 2 (EC2)- and C-terminal cytoplasmic Y196/200-dependent manners. We also showed that the CLDN6/SFK/PI3K/AKT cascade targets the AKT-phosphorylation sites in mouse retinoic acid receptor γ and human estrogen receptor α (ERα), and stimulates their activities.

CLDNs frequently show aberrant expression and/or subcellular localization in a wide variety of cancers, resulting in either promotion or repression of tumor progression [15,16,17,18,19,20,21,22], hypothetically due to dysregulated CLDN signaling. We previously reported that high CLDN6 expression in endometrial cancer is an independent prognostic factor that is significantly associated with several clinicopathological variables [23]. In a subsequent study, we uncovered that aberrant CLDN6 expression promotes endometrial cancer progression by hijacking the CLDN6–ERα axis [24]. We also found that aberrant CLDN6–ERα signaling contributes not only to cellular proliferation, but also to collective cell migration in the leading front of endometrial cancer cells.

Among CLDN subtypes, CLDN12 belongs to nonclassical CLDN members. CLDN12 is critical for vitamin D-dependent paracellular Ca^2+^ transport in the intestine and kidney [25,26,27,28]. On the other hand, upon searching the Cancer Genome Atlas (TCGA) database [29,30], *CLDN12* mRNA is highly overexpressed in diverse histological types of human cancer tissues, such as SCC and ADCA in a range of organs. However, the available anti-CLND12 antibodies, including ours [25,31], hamper the verification of the protein expression and function in normal and pathological tissues due to the insufficient specificities and applications [32]. Hence, an additional anti-CLND12 antibody with high selectivity and titer is absolutely prerequested to further study the nature of CLDN12.

In the present study, we developed a novel monoclonal antibody (mAb) that selectively recognizes human CLDN12 and works for immunohistochemistry of formalin-fixed paraffin-embedded (FFPE) tissues. Using this specific mAb, we show that the diminished CLDN12 expression is a poor prognostic biomarker for cervical cancer.

## 2. Results

### 2.1. Establishment of an Anti-Human/Mouse CLDN12 mAb

**Figure 1:**
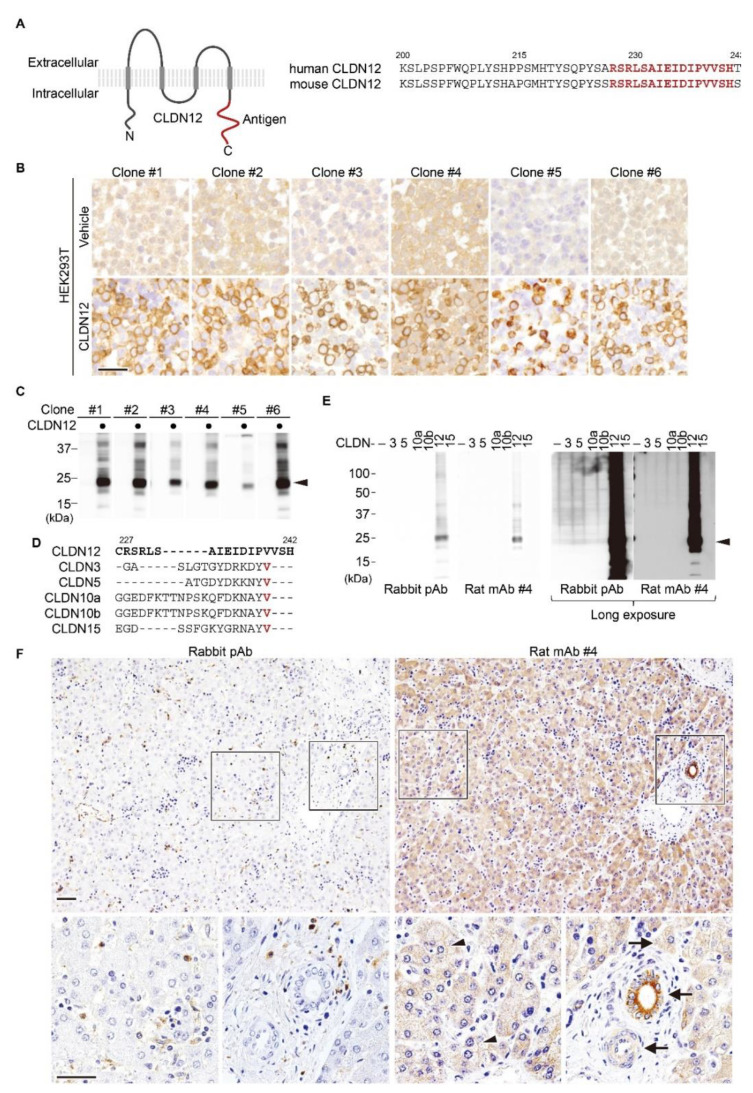
Generation of rat monoclonal antibodies (mAbs) against human/mouse claudin-12 (CLDN12). (**A**) Topology of CLDN12 (**left**) and amino acid sequences of the C-terminal cytoplasmic domains of human and mouse CLDN12 (**right**). The C-terminal region that correspond to an antigenic polypeptide is indicated in red. (**B**,**C**) HEK293T cells were transfected with the CLDN12 or empty expression vector, and cell blocks were subjected to immunohistochemical and Western blot analyses using the indicated anti-CLDN12 mAb clones. (**D**) Amino acid sequences of the antigenic peptide of the C-terminal cytoplasmic domain of human CLDN12 and the corresponding regions of the closely related CLDNs. Conserved amino acids are shown in red. (**E**) HEK293T cells were transfected with individual CLDN expression vector, and subjected to Western blot analysis using the indicated anti-CLDN12 Abs. (**F**) Normal human liver tissues were immunohistochemically stained with the indicated anti-CLDN12 Abs. Arrows and arrowheads reveal cytoplasmic and membranous signals, respectively. Scale bars, 100 μm.

We first generated a novel mAb against the same C-terminal cytoplasmic region between human and mouse CLDN12 (Figure 1A) using the medial iliac lymph-node method [33]. Among 202 hybridomas, 48 clones were selected by ELISA, six (clones #1/2/3/4/5/6) of which were able to detect positive signals by immunohistochemistry using cell block of CLDN12-expressing HEK293T cells (Figure 1B). Western blot analysis also revealed that these six clones reacted with human CLDN12 in HEK293T cells (Figure 1C).

Based on analysis using TCGA database, *CLDN12* mRNA is most abundantly overexpressed in colorectal cancer (Figure S2A). Therefore, we next validated the above-mentioned anti-CLDN12 mAbs by immunohistochemistry of colorectal cancer tissues, and selected clone #4 for further analyses.

To check the specificity of the rat anti-CLDN12 mAb (clone #4) and the formerly established rabbit anti-CLDN12 polyclonal antibody (pAb) [25,31], HEK293T cells were transiently transfected with distinct human CLDN expression vectors, followed by Western blot analysis. Both Abs selectively recognized CLDN12, but not CLDN3, CLDN5, CLDN10a/b or CLDN15, which are closely related to CLDN12 within the CLDN family (Figure 1D,E). Immunohistochemical analysis using clone #4 revealed that membranous and cytoplasmic CLDN12 signals appeared to be detected in hepatocytes of normal human liver tissues without lobular gradient (Figure 1F). In addition, CLDN12 is strongly expressed in portal cholangiocytes. Weak cytoplasmic CLDN12 signals were also observed in vascular smooth muscle cells, in good agreement with a previous report using CLDN12-lacZ-knockin mice [32]. Furthermore, CLDN12 was expressed in colorectal cancer tissues (Figure S2B). In marked contrast, by immunohistochemistry, the anti-CLDN12 pAb did not detect any specific signal in normal liver tissues or colorectal cancer tissues.

### 2.2. Expression of CLDN12 Protein in Normal, Premalignant and Malignant Tissues of the Uterine Cervix

**Figure 2:**
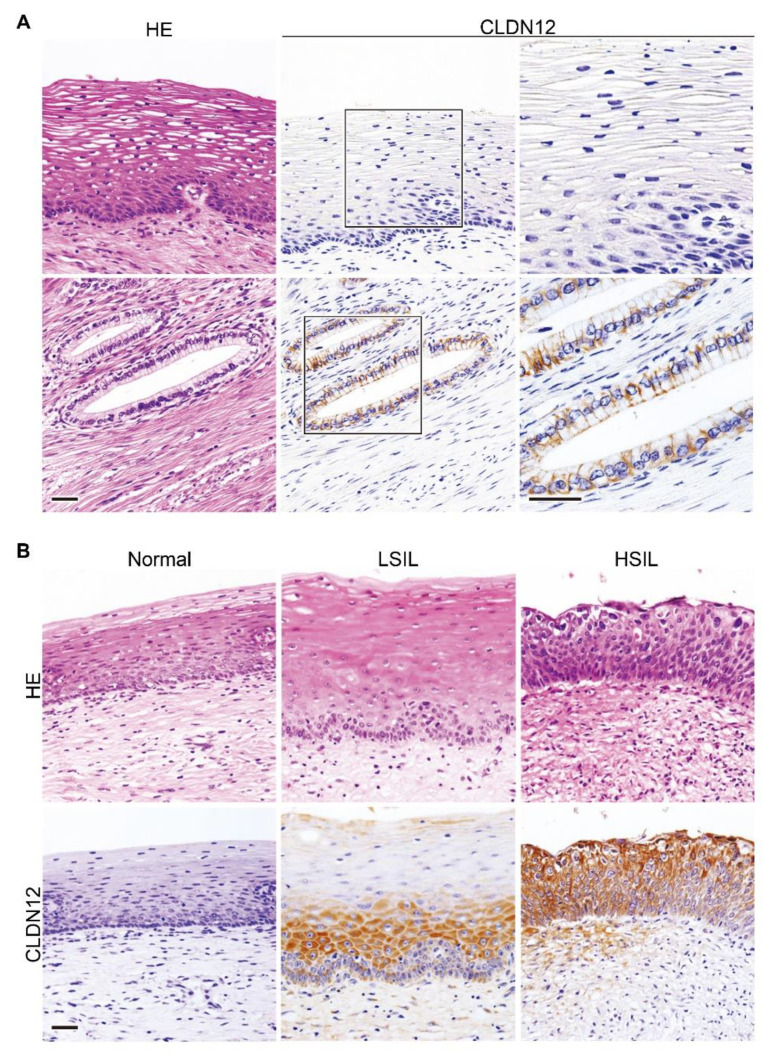
Expression of CLDN12 protein in normal and premalignant epithelial tissues of the uterine cervix. (**A**) Normal human cervical tissues and (**B**) squamous intraepithelial lesion (SIL) tissues were immunohistochemically stained with the anti-CLDN12 mAb. HE, hematoxylin-eosin; LSIL, low-grade SIL; HSIL, high-grade SIL. Scale bars, 100 μm.

We next determined by immunohistochemistry the CLDN12 expression in normal, premalignant and malignant epithelial tissues of the uterine cervix. As shown in Figure 2A, CLDN12 appeared to concentrate on cell membranes between normal cervical gland epithelia; on the other hand, it was not detected in squamous epithelial cells of the normal uterine cervix. Weak CLDN12 immunoreactivity was also observed in normal vascular and nonvascular smooth muscle cells in the uterus.

**Figure 3:**
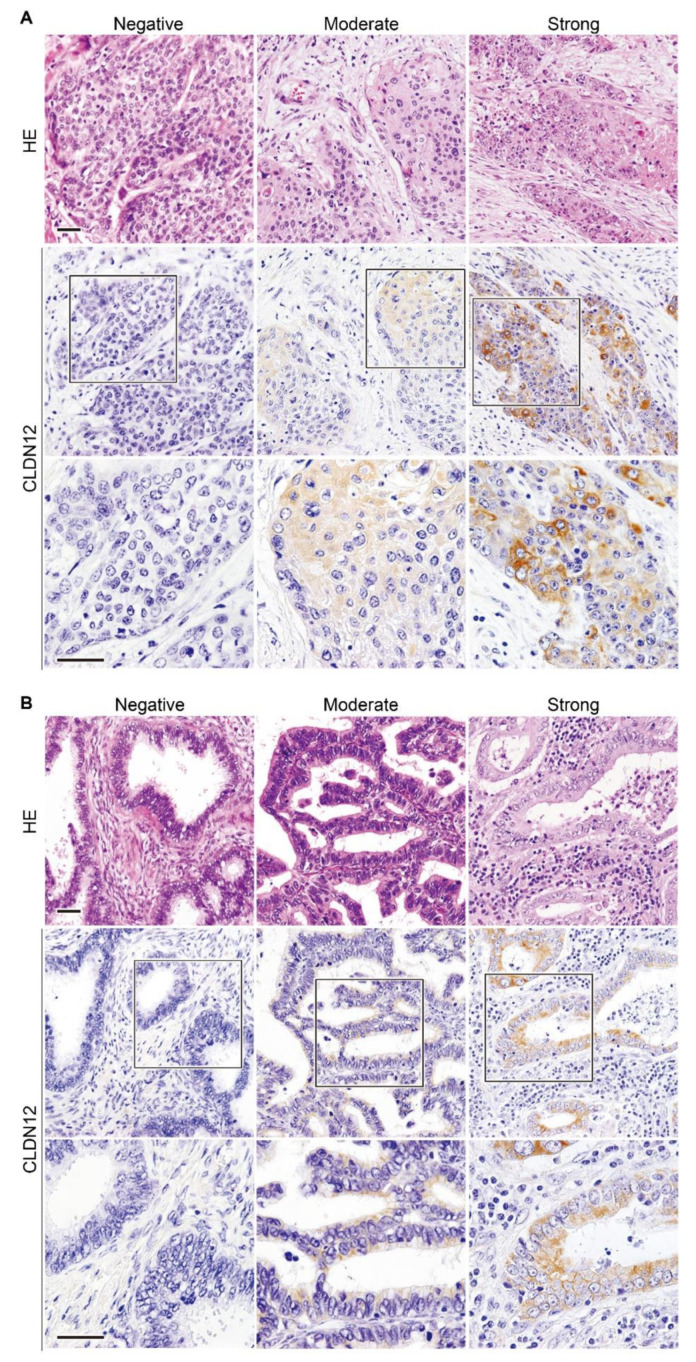
CLDN12 protein expression in cervical cancer tissues. (**A**) Squamous cell carcinoma (SCC) and (**B**) adenocarcinoma (ADCA) tissues were immunohistochemically stained with the anti-CLDN12 mAb. HE, hematoxylin-eosin. Scale bars, 100 μm.

Unlike normal squamous epithelial cells of the uterine cervix, CLDN12 was expressed throughout the cytoplasm of both low-grade squamous intraepithelial legion (LSIL) and high-grade SIL (HSIL) tissues (Figure 2B). CLDN12-immunoreactive signals were also observed in the cytoplasm of cervical SCC tissues, but the signal intensity (SI) appeared to be varied among the subjects (Figure 3A). By contrast, both intracellular and membranous signals for CLDN12 were detected in cervical ADCA tissues with the different SI (Figure 3B). By semiquantification of the CLDN12 expression in cervical cancer tissues, 26 of the 138 cases (18.8%) showed low CLDN12 expression, and the remaining 112 cases (81.2%) exhibited high CLN12 expression (Table 1).

### 2.3. Low Expression of CLDN12 Correlates with Poor Prognosis and Recurrence in Cervical Cancer

**Figure 4:**
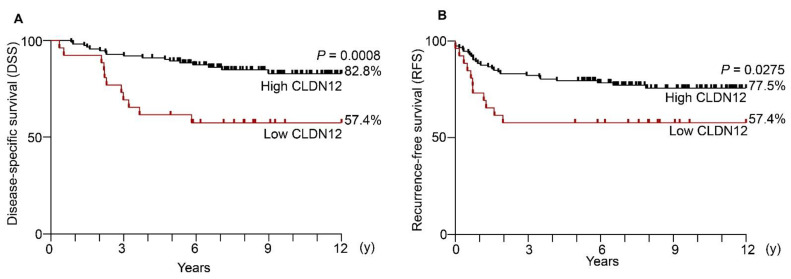
Low CLDN12 expression is associated with poor outcome in cervical cancer patients. (**A**) The disease-specific and (**B**) recurrence-free survival for low and high expression of CLDN12 protein in cervical cancer subjects are indicated.

Kaplan−Meier plots revealed significant differences in disease-specific survival (DSS) and recurrence-free survival (RFS) between the low and high CLDN12 expression groups (Figure 4A,B). The twelve-year DSS rates in the low and high CLDN12 groups were 57.4% and 82.8%, respectively.

Among the clinicopathological factors, the low CLDN12 expression was significantly associated with the recurrence of cervical cancer (*p* = 0.0385), but not with younger age, histological type, FIGO stage, tumor size, vascular involvement, lymphatic involvement, lymph node metastasis, distant metastasis or chemoradiotherapy (Table 1).

### 2.4. Reduced CLDN12 Represents an Independent Poor Prognostic Marker for Cervical Cancer

In the univariable analysis, the low CLDN12 expression (hazard ratio [HR] 3.412, *p* = 0.002), FIGO stage IIb/III/IV (HR 4.866, *p* < 0.001), tumor size (HR 3.945, *p* < 0.001), vascular involvement (HR 3.509, *p* = 0.005), lymphatic involvement (HR 2.973, *p* = 0.019), distant metastasis (HR 5.915, *p* = 0.004), chemoradiotherapy (HR 4.495, *p* = 0.041), and recurrence (HR 19.787, *p* < 0.001), showed significant prognostic variables for the DSS of cervical cancer patients (Table 2). By contrast, younger age, histological type, or lymph node metastasis were not prognostic markers for cervical cancer.

We subsequently performed multivariable analysis of the twelve-year DSS in cervical cancer subjects. Among the analyzed variables, the low CLDN12 expression (HR 2.615, *p* = 0.029), FIGO stage IIb/III/IV (HR 4.075, *p* = 0.002), and recurrence (HR 14.852, *p* < 0.001) were independent prognostic factors for the DSS of cervical cancer patients (Table 3).

## 3. Discussion

In the present study, we developed a novel rat anti-CLDN12 mAb (clone #4) that specifically reacted with CLDN12. Our immunohistochemical analysis of normal human liver FFPE tissues revealed that the anti-CLDN12 mAb was able to detect strong, moderate, and weak signals in portal cholangiocytes, hepatocytes and vascular smooth muscle cells, respectively. These results are in good agreement with previous findings using CLDN12-lacZ-knockin mice [32], ensuring a high reliability of our anti-CLDN12 mAb. When normal human uterus FFPE tissues were stained with the anti-CLDN12 mAb, CLDN12 immunoreactivity was observed in cervical gland epithelial cells. Taken together with the prominent selectivity of the novel anti-CLDN12 mAb, it could provide a powerful tool to determine expression and function of CLDN12 in a variety of normal and pathological tissues.

On Western blot and immunofluorescent analyses, the formerly generated anti-CLDN12 pAb selectively recognized mouse and human CLDN12 ([25,31]; this study). In the current study, however, we showed by immunohistochemistry that the anti-CLDN12 pAb, unlike the anti-CLDN12 mAb, detected no specific signal in normal liver or colorectal cancer FFPE tissues. In addition, we previously established anti-CLDN6 pAb [34] and anti-CLDN6 mAb [23]. On both Western blot and immunohistochemical analyses, the anti-CLDN6 mAb selectively recognized CLDN6, whereas the anti-CLDN6 pAb reacted not only with CLDN6, but also with overexpressed CLDN4 and CLDN5. Furthermore, it is known that certain anti-CLDN5 pAb recognizes both CLDN5 and CLDN6 [35]. Thus, it is of particular importance to verify the validity and cross-reactivity of the used anti-CLDN Abs depending on the applications.

Another issue that should be discussed is the subcellular localization of the CLDN12 protein in the normal and cancer tissues analyzed. The membranous CLDN12 signals appeared in uterine cervical epithelial cells, while the cytoplasmic signals were observed in portal cholangiocytes and smooth muscle cells. In hepatocytes, CLDN12 was distributed along cell membranes and throughout the cytoplasm. Both membranous and cytoplasmic CLDN12 signals were also observed in uterine cervical ADCA tissues. Moreover, in colorectal cancer and uterine cervical SCC tissues, CLDN12 was localized in the cytoplasm. Although we do not know why CLDN12 possesses distinct subcellular localization as described above, it has been reported that the CLDN1 localization is altered from cell membranes to cytoplasm at the invasion front of tongue SCC tissues [36].

Expression of CLDN1/2/4/7 proteins has been reported to be increased in cervical cancer tissues [37,38,39,40,41,42,43], but the clinicopathological significance has yet to be defined. We demonstrated in the current study that reduced CLDN12 expression predicts poor outcome in patients with cervical cancer. This conclusion was drawn from the following results: (1) the DSS and RFS in the low CLDN12 expression group of the cervical cancer subjects were significantly decreased compared with those in the high expression group; (2) the low CLDN12 expression was significantly associated with recurrence of cervical cancer; (3) upon univariable analysis, the low CLDN12 expression was found to be a significant prognostic variable for DSS of cervical cancer patients (HR 3.412, *p* = 0.002); (4) multivariable analysis revealed that the low CLDN12 expression was an independent prognostic factor for the DSS of cervical cancer subjects (HR 2.615, *p* = 0.029). Analysis of a larger number of cases would be required to obtain more solid conclusions about the clinicopathological relevance of the low CLDN12 expression in patients with cervical cancer.

It is unknown how the low CLDN12 expression contributes to poor prognosis in cervical cancer subjects. However, we have recently reported that aberrant CLDN6 signaling advances endometrial cancer progression via hijacking of the CLDN6–ERα pathway [24]. On the other hand, CLDN18 deficiency promotes tumorigenesis and progression of lung and gastric ADCA [44,45,46,47] through activating YES-associated protein (YAP) signaling. Along this line, the diminished CLDN12 expression may stimulate certain intracellular signaling pathways, such as YAP, resulting in cervical cancer progression.

## 4. Materials and Methods

### 4.1. Generation of Antibodies

A rabbit pAb against CLDN12 was generated in cooperation with Immuno-Biological Laboratories (#18801, Fujioka, Japan) as described previously [31].

Rat mAbs against CLDN12 were established using the iliac lymph node method [33]. In brief, a polypeptide, (C)RSRLSAIEIDIPVVSH, corresponding to the cytoplasmic domain of human and mouse CLDN12, was coupled via the cysteine to Imject Maleimide-Activated mcKLH (Thermo Fisher Scientific, Waltham, MA, USA). The conjugated peptide was intracutaneously injected with Imject Freund’s Complete Adjuvant (Thermo Fisher Scientific) into the footpads of anesthetized eight-week-old female rats. All animal experiments complied with the National Institutes of Health Guide for the Care and Use of Laboratory Animals, and were approved by the Animal Committee of Fukushima Medical University (FMU) (approval code, 2019-001; approval date, 1 April 2019). The animals were sacrificed 14 days after immunization, and the median iliac lymph nodes were collected, followed by extraction of lymphocytes by mincing. The extracted lymphocytes were fused with cells of the SP2 mouse myeloma cell line using polyethylene glycol. Hybridoma clones were maintained in GIT medium (Wako, Osaka, Japan) with supplementation of 10% BM-Condimed (Sigma–Aldrich, St. Louis, MO, USA). The supernatants were screened by enzyme-linked immunosorbent assay (ELISA).

### 4.2. Cell Culture, Expression Vectors and Transfection

HEK293T cells were grown in Dulbecco’s Modified Eagle Medium (DMEM, Glendale, AZ, USA) with 10% fetal bovine serum (FBS; Sigma–Aldrich) and 1% penicillin–streptomycin mixture (Gibco, Waltham, MA, USA).

The protein coding regions of human *CLDN3*, *CLDN5*, *CLDN10a*, *CLDN10b, CLDN12* and *CLDN15* were cloned into the *Bam*HI/*Not*I site of the CSII-EF-MCS-IRES2-Venus (RIKEN, RDB04384, Wako, Japan) plasmid.

For transient expression of the above-mentioned target genes, 5 × 10^6^ HEK293T cells were transfected with 10 µg of the indicated vectors using 30 µg of Polyethylenimine Max (PEI Max, Cosmo Bio, Carlsbad, CA, USA) 8 h after passage. Transfection efficiency was evaluated by Venus expression, with a fluorescent microscope (IX71, Olympus, Tokyo, Japan).

### 4.3. Immunoblotting

Total cell lysates were collected with CelLytic MT Cell Lysis Reagent (Sigma), followed by one-dimensional SDS-PAGE, and were electrophoretically transferred onto a piece of Immobilon (Millipore, Burlington, MA, USA). The membrane was saturated with PBS containing 4% skimmed milk and treated with primary antibodies. Supernatants of rat anti-CLDN12 hybridoma were directly used as primary antibodies, whereas rabbit anti-CLDN12 pAb was diluted at 1:2000 in PBS. The signal was detected by chemiluminescence using 1:2000-times diluted HRP-conjugated anti-rat IgG (NA935V, GE Health Care, Chicago, IL, USA) or anti-rabbit IgG (NA934V, GE Health Care).

### 4.4. Cell Blocks

Cells were centrifuged at 1200 rpm for 10 min and fixed with 10% formalin for 16 h at 4 °C. Fixed cell pellets were mixed with 1% sodium alginate followed by 1 M calcium chloride and embedded in paraffin (Tissue-Tek VIP 5 Jr, Sakura Finetek Japan, Tokyo, Japan).

### 4.5. Tissue Collection, Immunostaining and Analysis

FFPE tissue sections were obtained from: 138 patients with uterine cervical cancer (age, 29–79 years; average ± SD = 46.4 ± 12.0) who underwent hysterectomy alone or together with bilateral salpingo-oophorectomy and/or lymphadenoectomy between 2005 and 2015 at Fukushima Medical University Hospital (FMUH); and 37 patients with SIL subjects of the cervix; and four autopsy cases. Informed consent was obtained from all the patients or the next of kin for each subject. The cervical cancer subjects were limited to patients who were confirmed to have at least 5-year outcomes and those who had died due to cervical cancer and metastasis. Detailed information, including postoperative pathology diagnosis reports, age, histological type, stage (FIGO 2008), tumor size, vascular involvement, lymphatic involvement, lymph node metastasis, distant metastasis, chemoradiotherapy, recurrence status, DSS, and RFS, was obtained. The staging of patients between 2005 and 2007 was modified in accordance with the FIGO 2008 system. Distant metastasis was judged by diagnostic imaging. The study was approved by the Ethics Committee of FMUH (approval code, 2019-311; approval date, 18 March 2020).

For immunostaining, the 10% FFPE tissue blocks were sliced into 5-μm-thick sections, deparaffinized with xylene, and rehydrated using a graduated series of ethanol. The sections were then immersed in 0.3% hydrogen peroxide in methanol for 20 min at room temperature to block endogenous peroxidase activity. Antigen retrieval was performed by incubating the sections in boiling 10 mM citric acid buffer (pH 5.0) using a microwave. After blocking with 5% skimmed milk at room temperature for 30 min, the sections were incubated overnight at 4 °C with supernatants of the rat anti-CLDN12 hybridoma (1:4, clone #4) or the rabbit anti-CLDN12 pAb (1:100). After washing with PBS, a secondary antibody reaction was performed by using the Histofine mouse PO-Rat secondary antibody (Nichirei, Tokyo, Japan) or Histofine Simple Stain MAX-PO (MULTI) kit (Nichirei) with 3′,3′-diaminobenzidine (DAB) as a chromogen according to the manufacturer’s instructions.

Immunostaining results were interpreted by two independent pathologists and one gynecologist using a semiquantitative scoring system (immunoreactive score; IRS) [48]. The immunostaining reactions were evaluated according to SI (0, no stain; 1, weak; 2, moderate; 3, strong) and percentage of positive cells (PP: 0, <1%; 1, 1–10%; 2, 11–30%; 3, 31–50%; and 4, >50%). The SI and PP were then multiplied to generate the IRS for each case. To determine the optical cut-off values of IRS for CLDN12 expression, the receiver operating characteristic (ROC) curve was plotted and analyzed (Supplementary Figure S1). Based on this analysis, we divided the samples into two groups: low expression (IRS < 3) and high expression (IRS ≥ 3).

### 4.6. Statistical Analysis

We used the chi-squared test to evaluate the relationship between CLDN12 expression and various clinicopathological parameters. Survival analysis was performed using the Kaplan–Meier method, and differences between the groups were analyzed using the log-rank test. The Cox regression multivariable model was used to detect the independent predictors of survival. Two-tailed *p*-values < 0.05 were considered to indicate a statistically significant result. All statistical analyses were performed using GraphPad Prism 9 software (GraphPad Software, San Diego, CA, USA) and StaFlex ver.7 (Artech, Osaka, Japan).

## 5. Conclusions

In summary, we here established an mAb that selectively recognized CLDN12. We also demonstrated that the reduced CLDN12 expression was an independent prognostic variable for cervical cancer. The novel anti-CLDN12 mAb would be extremely valuable to determine the biological relevance of the CDLN12 expression in diverse diseases, including various types of cancer.

## Figures and Tables

**Table 1:** Relationship between CLDN12 expression and clinicopathological factors in cervical cancer.

Characteristics		Total (*n* = 138)		*p*-Value
		High CLDN12 (*n* = 112)	Low CLDN12 (*n* = 26)	
Age				
	<50	74 (66%)	15 (58%)	0.4964
	≥50	38 (34%)	11 (42%)	
Histological type				
	Adenocarcinoma	19 (17%)	1 (4%)	SCC vs. non-SCC 0.3533
	Adenosquamous carcinoma	14 (13%)	4 (15%)	
	Squamous cell carcinoma	73 (65%)	20 (77%)	
	Other	6 (5%)	1 (4%)	
FIGO Stage				
	I	71 (63%)	13 (50%)	I vs. II/III/IV 0.2653
	II	37 (33%)	12 (46%)	
	III	0 (0%)	0 (0%)	
	IV	4 (4%)	1 (4%)	
Tumor size				
	1	68 (61%)	12 (46%)	pT1 vs. pT2/3/4 0.1921
	2	43 (38%)	13 (50%)	
	3	0 (0%)	0 (0%)	
	4	0 (0%)	1 (4%)	
	Unknown	1 (1%)	0 (0%)	
Vascular involvement				
	No	60 (54%)	12 (46%)	0.5088
	Yes	47 (42%)	13 (50%)	
	Unknown	5 (4%)	1 (4%)	
Lymphatic involvement				
	No	51 (46%)	10 (38%)	0.6567
	Yes	57 (51%)	15 (58%)	
	Unknown	4 (4%)	1 (4%)	
Lymph node metastasis				
	N0	72 (64%)	17 (65%)	1.0000
	N1	32 (29%)	7 (27%)	
	NX	8 (7%)	2 (8%)	
Distant metastasis				
	M0	105 (94%)	25 (96%)	1.0000
	M1	4 (4%)	0 (0%)	
	MX	3 (3%)	1 (4%)	
Chemoradiotherapy				
	Chemotherapy	16 (14%)	4 (15%)	no vs. yes 0.7971
	Radiotherapy	29 (26%)	7 (27%)	
	Concurrent chemoradiotherapy	39 (35%)	10 (38%)	
	Other	1 (1%)	0 (0%)	
	None	27 (24%)	5 (19%)	
Recurrence				
	No	91 (81%)	16 (62%)	0.0385
	Yes	21 (19%)	10 (38%)	

**Table 2:** Univariable analysis of disease-specific survival in cervical cancer patients.

Variable		*p*-Value	Hazard Ratio	95% CI
CLDN12	Low	0.002	3.412	1.595–7.300
Age	≥50	0.603	0.810	0.366–1.792
Histological type	SCC	0.336	0.689	0.323–1.472
FIGO Stage	≥2b	<0.001	4.866	2.277–10.400
Tumor size	>4 cm	<0.001	3.945	1.862–8.360
Vascular involvement	Yes	0.005	3.509	1.465–8.409
Lymphatic involvement	Yes	0.019	2.973	1.193–7.408
Lymph node metastasis	Yes	0.355	1.452	0.659–3.199
Distant metastasis	Yes	0.004	5.915	1.780–19.66
Chemoradiotherapy	Yes	0.041	4.495	1.066–18.949
Recurrence	Yes	<0.001	19.787	7.969–49.135

CI, confidence interval.

**Table 3:** Multivariable analysis of disease-specific survival in cervical cancer patients.

Variable		*p*-Value	Hazard Ratio	95% CI
CLDN12	Low	0.029	2.615	1.101–6.214
FIGO Stage	≥2b	0.002	4.075	1.679–9.891
Recurrence	Yes	<0.001	14.852	5.847–37.726

CI, confidence interval.

## Data Availability

The data presented in this study are available on request from the corresponding author.

## Supplementary Materials

The following are available online at https://www.mdpi.com/article/10.3390/ijms22073774/s1.
