# Supplementary material for: Reduced Claudin-12 Expression Predicts Poor Prognosis in Cervical Cancer
Source: Int J Mol Sci. 2021 Apr 6;22(7):3774. doi: 10.3390/ijms22073774 (PMC8038723; doi:10.3390/ijms22073774)
Supplement: Supplementary file 1 [file ijms-22-03774-s001.pdf]

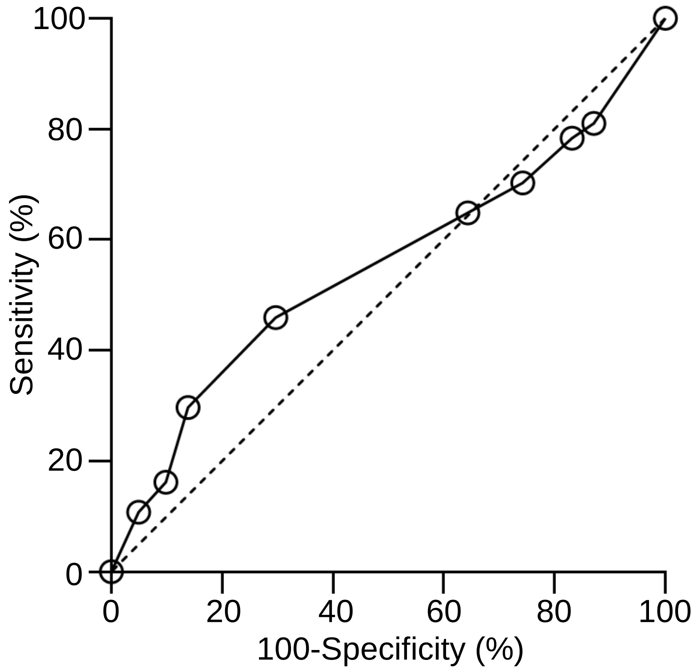

IRS cut off	Sensitivity%	95% CI	Specificity%	95% CI	Likelihood ratio
< 0.5000	10.81	4.285% to 24.71%	95.05	88.93% to 97.87%	2.184
< 1.500	16.22	7.651% to 31.14%	90.1	82.73% to 94.53%	1.638
< 2.500	29.73	17.49% to 45.78%	86.14	78.07% to 91.56%	2.145
< 3.500	45.95	31.04% to 61.62%	70.3	60.77% to 78.33%	1.547
< 5.000	64.86	48.76% to 78.17%	35.64	26.99% to 45.35%	1.008
< 7.000	70.27	54.22% to 82.51%	25.74	18.22% to 35.05%	0.9463
< 8.500	78.38	62.80% to 88.61%	16.83	10.78% to 25.31%	0.9424
< 10.50	81.08	65.80% to 90.52%	12.87	7.679% to 20.78%	0.9306

Figure S1. The receiver operating characteristic (ROC) curves for disease specific survival using cutoff score of CLDN12-immunoreactive score (IRS) ≧ 3. CI, confidence interval.

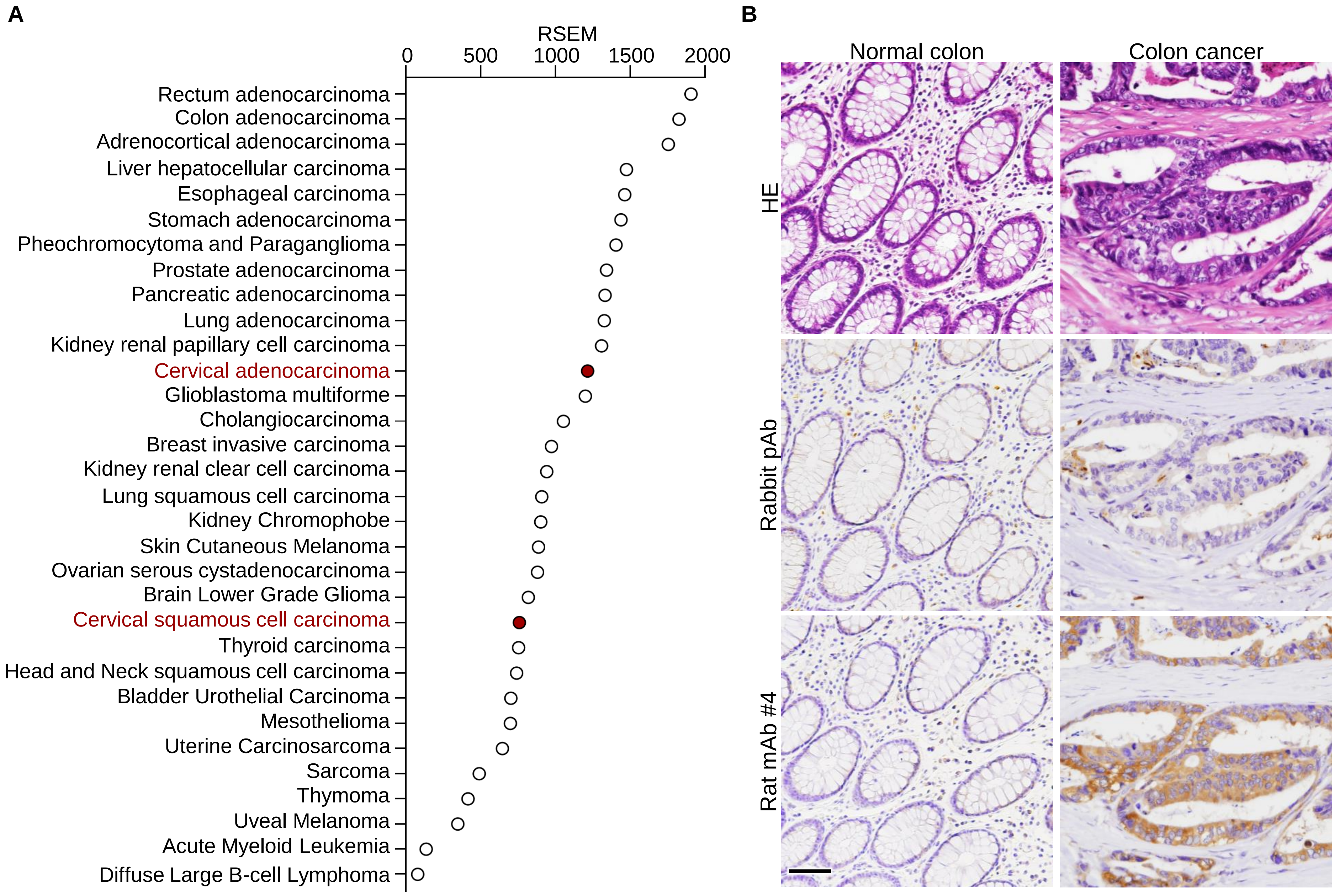

Figure S2. The *CLDN12* mRNA expression in various cancer tissues and the CLDN12 protein expression in normal and malignant colon tissues. (**A**) Expression levels of *CLDN12* mRNA in the indicated cancer tissues were analysed using TCGA database. RSEM, RNA-Seq by Expectation-Maximization. (**B**) Normal and cancer tissues of the colon were immunohistochemically stained with the anti-CLDN12 pAb and the anti-CLDN12 mAb. HE, hematoxylin-eosin. Scale bar, 100 μm.
